# Medical Legislation—the Quarantine Bill

**Published:** 1891-04

**Authors:** 


### EDITORIAL DEPARTMENT.

F. E. DANIEL, M. D., EDITOR AND PUBLISHER.

THIS JOURNAL, by unanimous vote of the members, was made the Official Organ of the Austin District Medical Society.

COLLABORATORS:

*Dr. R. M. Swearingen, Austin.*
*Prof. B. E. Hadra, M. D., Galveston.*
*Prof. Geo. Cupples, M. D., San Antonio.*
*Dr. T. C. Osborn, Cleburne, Texas.*
*Dr E. J. Doering, Chicago.*
*Dr. E. J. Beall, Fort Worth.*
*Dr Odo Betz, Germany.*
*Prof. W. B. Rogers, M. D., Memphis.*
*Dr. J. W. McLaughlin, Austin.*
*Dr. T. J. Tyner, Austin.*
*Prof. J. F. Y. Paine, M. D., Galveston.*
*Dr. R. H. L. Bibb, Mexico.*
*Dr. T. J. Bennett. Austin.*
*Dr. Bat Smith, Wharton.*
*Dr. E. Meierhof, New York.*
*L. H. Luce, M. D., West Tisbury, Mass.*

### MEDICAL LEGISLATION—THE QUARANTINE BILL.

In our last issue it was announced that the bill to regulate the practice of medicine, prepared by the Association's committee had been killed in the Senate by amendments; that it had then been indefinitely postponed, and in a P. S. it was announced that a motion to reconsider the motion postponing it indefinitely ha prevailed, and the bill was again "up in the Senate." The latter statement was a little premature. The motion was reconsidered —thus restoring the bill to its former status—liable *to be* brought up again. But since that time nothing has been done. So much legislation of another character was pressing that it has not been possible for the friends of the bill to get a hearing. Meantime, a friendly Senator, at the suggestion of friends of the medical bill, re-enacted the penal statute relating to practice, as it appears on the books, omitting the line or clause which, in 1876, was inserted by the friends of the opposition, which clause exempted from examination all holders of diplomas. This is the clause or amendment which, it is understood by the profession, operated to emasculate the bill. Of course, there are other defects; too many boards; boards appointed by county judge, etc.; but it was thought if we could get the present law so amended as to make it operative, by defining the offense of practicing medicine without license, (which had been omitted in the original draft), and by striking out the clause referred to above, it would be as much as could be expected at present, and would, really, give us a very good law. But, to date, our Senator friend has not had opportunity to get his bill before the Senate. So, we may say, medical legislation (or the lack of it) remains, at this writing, *statu quo.*

However, something has been done looking to improving our present health laws. Representative McKinney, of Walker county, with the aid of our very popular health officer, Dr. Swearingen, has prepared a bill,* which, in effect, organizes internal quarantine. Our present law provides protection against invasion by land or by sea, but heretofore there has been no provision for internal sanitation—no understanding as to what is to be done in case of small-pox, diphtheria, cholera, etc., breaking out in the interior. This bill, as will be seen, organizes health authorities in each county and city, and makes them subordinate to the State Health Officer.

As far as it goes, if passed, the bill will do very well; it provides for a necessity long felt, and, doubtless, it will work well, as the subject has been long and seriously thought of and studied by the State Health Officer. And, inasmuch as the Commissioner of Statistics and Agriculture is authorized to secure and preserve vital statistics—this bill, if passed—may supercede the necessity for a State board of health; and the expense of operating it, added to that of the quarantine as it stood, will be, perhaps, less than the cost of a bran new board of health. At any rate, it is the best that can be done at present, as the people and the legislators—judging alone by the fact that Texas has had no epidemics in years—are wedded to the present law, and could not easily be induced to give it up and try a new one. As will be seen, also, the expense, in case of outbreaks in the interior, does not fall entirely on the State, which is very proper. It has always been the JOURNAL'S pet idea, in view of the objections pointed out above, to enlarge upon the quarantine law, so as to make it embrace vi-

---

*Published in this number of the JOURNAL.

tal statistics and internal sanitation, in place of attempting to create a State Board *de novo*.

This bill will probably be acted upon in the House, ere this reaches our readers; but whether it will make the round trip before adjournment, or not, is just one of those things which cannot be foreseen. We shall see.

P. S. The bill passed the House on the 10th inst., with only nine dissenting votes.

